# Repurposing Antispasmodic Agent Otilonium Bromide for Treatment of *Staphylococcus aureus* Infections

**DOI:** 10.3389/fmicb.2020.01720

**Published:** 2020-07-31

**Authors:** Linying Zhou, Pengfei She, Fang Tan, Shijia Li, Xianghai Zeng, Lihua Chen, Zhen Luo, Yong Wu

**Affiliations:** Department of Laboratory Medicine, The Third Xiangya Hospital, Central South University, Changsha, China

**Keywords:** otilonium bromide, *Staphylococcus aureus*, antibacterial, biofilm, membrane permeability

## Abstract

Recently, the problem of bacterial resistance has been brought into focus, which makes the development of new antibiotics become a necessity. Compared with traditional development approaches, drug repurposing provides a faster and more effective approach to find new antimicrobial agents. In this study, we found that antispasmodic agent otilonium bromide had strong antibacterial ability and bactericidal activity against *Staphylococcus aureus*, with minimal inhibitory concentrations (MICs) of 4–8 μg/ml, and bacteria could be killed completely after treatment with 2× MIC of otilonium bromide for 5 h. Furthermore, it had a potent effect on eradicating biofilm at concentrations ranging from 16 to 64 μg/ml. At the same time, it had low tendency to develop resistance and possessed limited cytotoxicity. In the methicillin-resistant *S. aureus*–infected mouse peritonitis model, it was also effective to cure mice and improve their survival rate. In addition, we observed that otilonium bromide changed the permeability of bacterial membrane and caused membrane damage, and it is probably the antibacterial mechanism of otilonium bromide. Taken together, our results indicated that otilonium bromide could be a new antimicrobial agent to treat *S. aureus* infections more safely and efficiently.

## Introduction

*Staphylococcus aureus* is a common pathogen that can cause hospital-acquired infections, and its isolation rate was very high among Gram-positive bacteria (Gould et al., 2012). It can cause various diseases such as pneumonia, catheter-related infections, and sepsis (David and Daum, 2017). In addition, owing to the overuse of antibiotics, the problem of bacterial resistance has become more and more serious. The detection rate of methicillin-resistant *S. aureus* (MRSA) has risen continuously, which poses a huge challenge to clinical treatment. Therefore, how to treat various diseases caused by *S. aureus* efficiently and economically has become an urgent problem.

Recently, greater attention has been paid to drug repurposing which is a strategy to explore new antimicrobial agents. Most of the drugs discovered through this approach have detailed information about safety and pharmacokinetic profiles, which reduces the cost and time for developing new drugs and accelerates its application in clinical treatment (Miró-Canturri et al., 2019).

**FIGURE 1:**
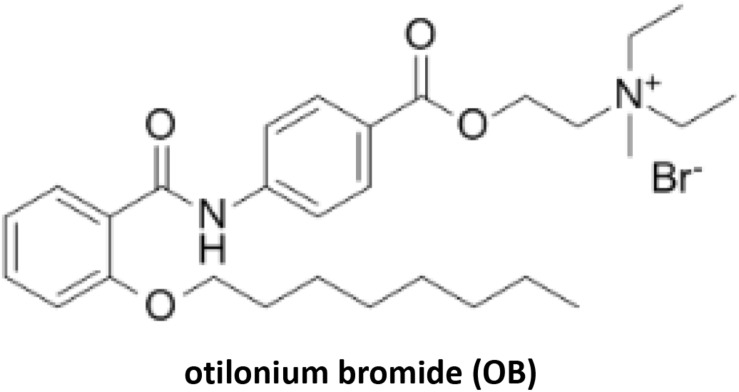
Chemical structure of otilonium bromide.

Quaternary amine compound otilonium bromide (OB) (Figure 1) is a FDA-certified antispasmodic agent which is commonly used in the treatment of irritable bowel syndrome (IBS). The main mode of action is blocking the L-type Ca^2+^ channels on smooth muscles and interfering with intracytoplasmatic Ca^2+^ mobilization, thus relieving excessive intestinal contractions and abdominal pain (Evangelista et al., 2018). It has been reported that OB has potential antibacterial activity against multidrug-resistant *Acinetobacter baumannii* and *S. aureus* by damaging cell membrane and disrupting cellular protein homeostasis (Knauf et al., 2018). However, there has been no systematic study to evaluate its antibacterial property, toxicity, and *in vivo* efficacy. Here, evaluating the antibacterial activity of OB *in vitro* and *in vivo*, we believe that OB has a possibility to be used as a novel antibacterial agent for the treatment of staphylococcal infections.

## Materials and Methods

### Bacterial Strains and Growth Conditions

Ten clinical isolations of *S. aureus* were collected from patients admitted in the Third Xiangya Hospital of Central South University. *S. aureus* Newman and ATCC 43300 were provided by Min Li (Shanghai Jiao Tong University, Shanghai, China). *Staphylococcus epidermidis* RP62A (ATCC 35984) was provided by Di Qu (Fudan University, Shanghai, China). *Pseudomonas aeruginosa* PAO1 (ATCC 15692) was obtained from Mingqiang Qiao (Nankai University, Tianjin, China). *Enterococcus faecalis* ATCC 29212, *A. baumannii* ATCC 1195, *Klebsiella pneumoniae* ATCC 700603, *Escherichia coli* ATCC 25922, and *S. aureus* ATCC 29213 were purchased from the American Type Culture Collection. *S. aureus* and *S. epidermidis* strains were grown in tryptic soy broth (TSB) (Solarbio, Beijing, China) medium. *E. faecalis* strain was cultured in brain–heart infusion broth (Solarbio) medium. Other strains were grown in Luria–Bertani broth (Solarbio) medium.

### Reagent Preparation

Oxacillin, vancomycin, OB, melittin, and polymyxin B nonapeptide (PMBN) were purchased from MedChem Express (NJ, United States). Ciprofloxacin and rifampin were purchased from Aladdin (Shanghai, China). Oxacillin, vancomycin, ciprofloxacin, and melittin were prepared in deionized water at 6.4, 25, 6.4, and 1 mg/ml, respectively. OB, PMBN, and rifampin were prepared in DMSO at 100, 10, and 10 mg/ml, respectively.

### Antibacterial Assays

The minimal inhibitory concentration (MIC) was determined by microdilution method according to Clinical and Laboratory Standards Institute Guidelines (Harbut et al., 2015). After determining the MIC, 5 μl of bacterial cultures was taken from wells in which the drug concentration was equal to or higher than the MIC, and they were plated on blood agar plates. The minimum bactericidal concentration (MBC) was defined as the lowest concentration for which no bacterial colonies were grown on plates after incubation for 24 h at 37°C (Tan et al., 2019). All experiments were repeated in triplicate.

### Time-Kill Assay

An overnight culture of *S. aureus* ATCC 43300 and ATCC 29213 was adjusted to cell concentration of 1 × 10^6^ CFU/ml in Mueller–Hinton broth (MHB) (Solarbio) medium and was incubated with 0.5× MIC, 1× MIC, 2× MIC, and 4× MIC of OB or without drug. The cultures were incubated at 37°C with shaking at 180 rpm. Samples were taken at 0, 1, 2, 3, 4, 5, 6, 12, and 24 h, then diluted and plated on blood agar plates. After incubation at 37°C for 24 h, colonies were counted and viable cells were determined (CFU/ml) (Thakare et al., 2017). The experiment was repeated in triplicate.

### Checkerboard Assay

The bacterial suspension at mid-log phase was adjusted to 1 × 10^6^CFU/ml and dispensed into 96-well microtiter plates (Corning/Costar, United States), and then a two-dimensional checkerboard with serial dilutions of OB and PMBN (ranging from 1 to 128 μg/ml) were set up. The results were detected by a microplate reader (iMark Microplate Absorbance Reader; BIO-RAD, United States) at OD_630 *nm*_ after incubation at 37°C for 18–24 h. The experiment was repeated in triplicate and the fractional inhibitory concentration index (FICI) was calculated by using the following formula:

$$\text{FICI}=\frac{{\text{MIC}}_{\text{A}}\,\,\left(\text{combination}\right)}{{\text{MIC}}_{\text{A}}\,\,\left(\text{alone}\right)}+\frac{{\text{MIC}}_{\text{B}}\,\,\left(\text{combination}\right)}{{\text{MIC}}_{\text{B}}\,\,\left(\text{alone}\right)}$$

Classification criteria: FICI ≤ 0.5 is synergistic; 0.5 < FICI ≤ 1 is additive; 1 < FICI ≤ 4 is irrelevant; >4 is antagonistic (Pengfei et al., 2020).

### Anti-biofilm Assays

All *S. aureus* strains including 10 clinical strains were inoculated into TSB medium with 1% glucose and cultured at 37°C for 24 h, with shaking at 180 rpm. Then 200 μl of bacterial suspension (100-fold dilution) was added to 96-well microtiter plates to form biofilm. After incubation at 37°C for 24 h without shaking, the plates were washed with PBS to remove planktonic cells, stained with 0.5% crystal violet for 15 min, and washed with PBS to remove excess dye. Then the stained dye was dissolved with 200 μl of 95% alcohol for 20 min. The absorbance (A_570 *nm*_) was measured in a microplate reader and the biofilm-forming capacity of all *S. aureus* strains was classified according to the criteria as previously reported (Supplementary Table S1; Hassan et al., 2011). Subsequently, nine strains that were classified as strong biofilm producers were selected to form biofilm as described previously. After 24 h, the medium was discarded and the plates were washed. Biofilm was treated with drugs (ranging from 4 to 128 μg/ml) in 200 μl of TSB medium for 24 h at 37°C. As a control, biofilm was exposed to TSB medium without drug. Finally, the plates were washed again and stained with 0.5% crystal violet. MBEC was defined as the lowest drug concentration of drug which eradicated biofilm by 50% (MBEC_50_), relative to the drug-free control well (Melo et al., 2011). All experiments were repeated in triplicate.

### qPCR

Briefly, *S. aureus* ATCC 43300 was cultured in a 6-well microtiter plate and treated with or without OB for 24 h. The total RNA was extracted using E.Z.N.A. Bacterial RNA Kit (Omega, United States), cDNA was prepared using TransScript All-in-One First-Strand cDNA Synthesis SuperMix (Transgene, Beijing, China), and qPCR was performed with TransStart Tip Green qPCR SuperMix (Transgene) using a CFX96 Real-Time PCR Detection System (Bio-Rad Laboratories, United Kingdom) (94°Cfor 5 s, 40 cycles of 94°C for 5 s, 58°C for 15 s, and 72°C for 10 s). Primers were used as follows: *icaA* forward, ACACTTGCTGGCGCAGTCAA; *icaA* reverse, TCTGGAACCAACATCCAACA; *icaD* forward, ATGGTCAAGCCCAGACAGAG; and *icaD* reverse, AGTATTTTCAATGTTTAAAGCAA (Haddad et al., 2018). The primer 16S RNA was used as an internal standard and the experiment was repeated in triplicate.

### Confocal Laser Scanning Microscope

The efficiency of OB in eradicating biofilm was visually assessed by confocal laser scanning microscope (CLSM). Briefly, an overnight culture of MRSA ATCC 43300 and SA 1420 (clinical strain) was diluted 1:100 with TSB medium, and 2 ml of bacterial suspension was added to a 6-well microtiter plate. Next, sterile glass slides were placed into the plate to form mature biofilm. After incubation at 37°C for 24 h without shaking, the slides were washed with PBS, treated with 4× MIC or 8× MIC of OB, and incubated for another 24 h. As a control, the slides were not treated with drug. The slides were then washed again, stained with SYTO9 fluorescence dye (Thermo Fisher Scientific, United States) according to the manufacturer’s instructions, and observed by CLSM (Zeiss LSM800, Germany). The biofilm quantification was performed with ZEN 3.0 (blue edition) software (She et al., 2019).

### Resistance Selection

Single-step resistance selection and multi-step resistance selection were applied to evaluate the drug resistance of *S. aureus* to OB. For single-step resistance selection, an overnight culture of *S. aureus* ATCC 43300 and ATCC 29213 was prepared in TSB medium to an OD_630 *nm*_ of 0.5. Then, 100 μl of diluted bacterial cultures was plated on MH agar (without drug or with 2× MIC, 4× MIC of ciprofloxacin, rifampicin, and OB) to quantitate the starting inoculum or resistant colonies. After incubation at 37°C for 48 h, the resistance frequency was calculated as the number of drug-resistant mutants divided by the number of total colonies (Trombetta et al., 2018). For multi-step resistance selection, on the first day, the MICs of OB and ciprofloxacin against *S. aureus* ATCC 43300 and ATCC 29213 were determined as previously described. After incubation for 16–18 h, 5 μl of bacterial cultures was taken from wells where the drug concentration was 0.5× MIC and was diluted 1000-fold with MHB medium. Afterward, 50 μl of bacterial suspension and 50 μl of medium containing a serially diluted drug were added to 96-well microtiter plates for the next-day MIC assay, and the protocol was repeated 20 times (Friedman et al., 2006). All experiments were repeated in triplicate.

### Membrane Permeability Assays

The effect of OB on the bacterial membrane of *S. aureus* was assessed by two fluorescence dyes, SYTOX Green and Disc3(5). Nucleic acid stain SYTOX Green could not cross over intact membrane, but it could penetrate through compromised membrane and combine with nucleic acid to exhibit bright green fluorescence (>500-fold fluorescence enhancement). Cationic dye Disc3(5) could detect the membrane depolarization and increase the fluorescence signal (Roth et al., 1997; Miyazaki et al., 2017). Briefly, *S. aureus* ATCC 43300 and ATCC 29213 in mid-log growth-phases were suspended in 5 mM HEPES (pH 7.2) with an OD_630__*nm*_ of 0.05. The bacterial suspension was incubated with 2 μM SYTOX Green (Thermo Fisher Scientific, United States) in dark for 15 min and then was treated with serially diluted OB. The fluorescence intensity was detected by a microplate reader (PerkinElmer EnVision, United States) for 30 min at the excitation wavelength of 504 nm and emission wavelength of 523 nm (Koh et al., 2013; Lee et al., 2013). The detection of membrane depolarization was slightly different. The bacterial suspension was incubated with 5 mM glucose, 100 mM KCl, and 2 μM Disc3(5) (AAT Bioquest, United States) in dark for 1 h, at which time different concentrations of OB were added and the fluorescence intensity monitored every 30 s for 5 min (excitation wavelength 622 nm; emission wavelength 670 nm) (Miyazaki et al., 2017). Melittin (20 μg/ml) was used as a positive control, and HEPES with 0.1% DMSO was used as a negative control. All experiments were repeated in triplicate.

The β-galactosidase assay was used to detect the inner membrane integrity of *E. coli*. If the cell membrane was destroyed, *ortho*-nitrophenol-β-galactoside (ONPG) (Sigma-Aldrich, United States) could enter the cytoplasm and be converted to yellow compound *O*-nitrophenol by reacting with β-galactosidase produced by *E. coli*. Briefly, *E. coli* ATCC 25922 in mid-log growth phase was suspended in PBS with an OD_405 *nm*_ of 1.2. Thereafter, 100 μl of bacterial suspension, 50 μl of 5 mM ONPG, and 50 μl of OB (0.5× MIC, 1× MIC, and 2× MIC) or DMSO (negative control) were added to the 96-well microtiter plate. The time-dependent color changes of product were monitored by a microplate reader at 405 nm every 10 min for 1.5 h (Han et al., 2016).

### Transmission Electron Microscopy

*S. aureus* ATCC 43300 in mid-log growth phase was suspended in PBS to 2 × 10^9^CFU/ml and was treated with 8× MIC of OB for 1 h at 37°C. As a control, bacteria were not treated with drug. The bacterial suspension was centrifuged at 4000 × *g* for 5 min to harvest the cell pellets, then the specimens were observed by transmission electron microscope (HITACHI HT7700, Japan) as previously mentioned (de Breij et al., 2018).

### Hemolysis Assay and Cytotoxicity Test

Blood samples were collected from a healthy individual at the Third Xiangya Hospital of Central South University and centrifuged at 1000 × *g*, 4°C for 5 min to harvest the human red blood cells (RBCs). RBCs were washed with PBS three times and suspended in PBS. Thereafter, RBC suspension (100 μl) and serially diluted OB (100 μl) were added to the 96-well microtiter plate to make the final RBC concentration of 4% (*v*/*v*). After incubation at 37°C for 1 h, 100 μl of supernatant was taken and the absorbance (A_570 *nm*_) was detected. In addition, 0.1% DMSO served as a negative control, 0.1% Triton X-100 was used as a positive control (Gerits et al., 2017). The hemolysis rate was calculated by using the following formula:

$$\text{Hemolysis}=\frac{{\text{A}}_{\text{sample}}-{\text{A}}_{0.1\%\,\mathrm{DMSO}}}{{\text{A}}_{0.1\%\,\mathrm{TritonX}-100}-{\text{A}}_{0.1\%\,\mathrm{DMSO}}}\times 100\%$$

The Cell Counting Kit-8 (DojinDo, Japan) was used to test the cytotoxicity of OB on human breast cancer cell line KPL-4 and human colon epithelial cell NCM-460. KPL-4 cells were cultured with DMEM medium containing 10% fetal bovine serum, and NCM-460 cells were grown in RPMI-1640 medium supplemented with 10% fetal bovine serum. Briefly, cells (100 μl) were seeded into 96-well microtiter plates at 4000 cells/well and cultured at 37°C with 5% CO_2_ for 24 h to make them adhere. Then, the supernatant was discarded and cells were treated with 100 μl of serially diluted OB or 0.1% DMSO for another 24 h. Finally, 10 μl of CCK-8 was added to each well and the absorbance (A_450__*nm*_) was recorded after 3 h (Tan et al., 2019). The cell viability was calculated according to the following formula:

$$\text{Viability}=\frac{{\text{A}}_{\text{sample}}-{\text{A}}_{\text{blank}}}{{\text{A}}_{0.1\%\,\mathrm{DMSO}}-{\text{A}}_{\text{blank}}}\times 100\%$$

All experiments were repeated in triplicate.

### Mouse Peritonitis Model and *in vivo* Toxicity

All animal studies were conducted under the approval of the Ethics Committee of the Third Xiangya Hospital, Central South University. The mice used in the experiment were female ICR, 24–26 g, purchased from Hunan SJA Experimental Animal Co. Ltd. (Changsha, China). Mice were maintained on a 12:12 light/dark cycle at 22–24°C in polycarbonate cages. Adequate water and food were provided in accordance with the relevant requirements of animal ethics. For *in vivo* toxicity determination, the mice (*n* = 5) were intraperitoneally injected twice (4-h interval) with 40 mg/kg of OB or DMSO (vehicle) and observed for 7 days. In addition, 40 mg/kg of OB or DMSO (vehicle) was injected (i.p.) into mice (*n* = 5) daily for 7 days and the mice were observed for 5 days after the final injection. On the 12th day, the mice were sacrificed and their blood and tissues were harvested for blood cells analysis and histological analysis (H&E staining). The MRSA-infected mouse peritonitis model was established as previously described (Gómez-Zorrilla et al., 2017; Su et al., 2019). Briefly, the mice were intraperitoneally injected with 500 μl of (2–3) × 10^8^ CFU/ml bacterial suspension (*S. aureus* ATCC 43300) containing 5% mucin (Cool Chemistry, Beijing, China). Then, groups of mice (*n* = 5) were given 30 mg/kg of OB, 40 mg/kg of OB, 50 mg/kg of vancomycin (positive control), or saline with 2% DMSO (vehicle) at 1 and 5 h post-infection through intraperitoneal injection. The survival condition and weight changes of the mice were observed continuously for 7 days. The survived mice were euthanized on the seventh day and their liver and kidney were homogenized and plated on blood agar plates to measure the number of viable bacteria.

## Results

### Antimicrobial Activities of OB Against *S. aureus*

The results of the MIC and MBC assays are shown in Table 1. *S. aureus* ATCC 43300 (MRSA) and four clinical isolates of MRSA were resistant to oxacillin (MICs ≥ 64 μg/ml; MBCs ≥ 128 μg/ml). All strains were sensitive to vancomycin, with MICs of 0.25–1 μg/ml and MBCs of 0.5–8 μg/ml. In addition, the spasmolytic agent OB also showed antimicrobial activity against *S. aureus*, with MICs of 4–8 μg/ml and MBCs of 8–16 μg/ml, as previously reported (Knauf et al., 2018).

**TABLE 1:** Antimicrobial and anti-biofilm activities of OB against *S. aureus*.

**Strains**	**Oxacillin (μg/ml)**			**Vancomycin (μg/ml)**			**OB (μg/ml)**		
	**MIC**	**MBC**	**MBEC_50_**	**MIC**	**MBC**	**MBEC_50_**	**MIC**	**MBC**	**MBEC_50_**
ATCC 43300^*ab*^	64	>128	>128	0.5	2	>128	8	16	32
SA 1409^*a*^	>128	>128	/	0.5	0.5	/	4	8	/
SA1417^*ab*^	>128	>128	>128	0.25	2	>128	8	8	32
SA1420^*ab*^	>128	>128	>128	0.5	2	>128	8	16	16
SA1430^*ab*^	>128	>128	>128	0.5	1	>128	4	16	64
SA 1402^*b*^	4	4	>128	1	1	16	8	8	16
SA 1405^*b*^	2	4	>128	1	8	16	8	8	32
SA 1411^*b*^	0.5	1	>128	0.5	4	>128	4	8	64
SA 1415^*b*^	1	4	32	0.5	8	16	4	8	16
SA 1419	0.5	16	/	1	4	/	8	16	/
LZ B1^*b*^	0.5	8	>128	0.5	1	>128	8	16	64
ATCC 29213	0.25	16	/	1	1	/	8	16	/
Newman	0.25	16	/	0.5	2	/	4	8	/

**“a” means the strain is MRSA; “b” means the strain is a strong biofilm producer; “/” means no measurement.**

**FIGURE 2:**
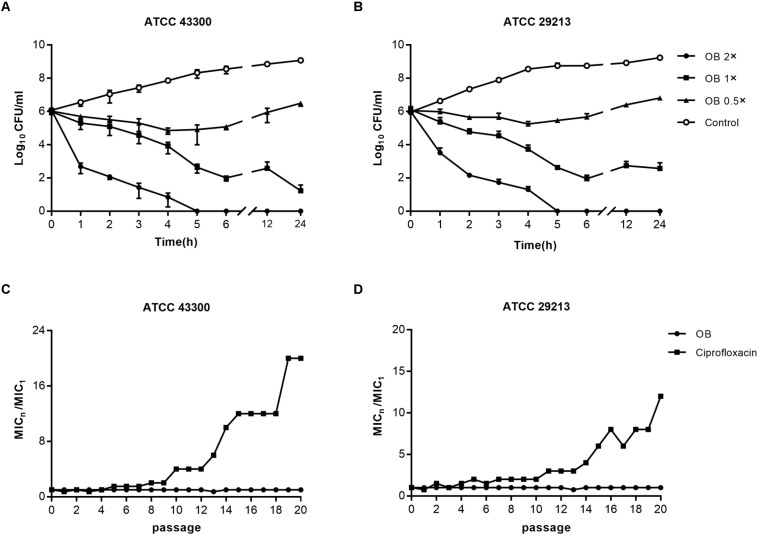
The bactericidal activity of OB against *S. aureus* and resistance development of *S. aureus* to OB. Time-kill assays of OB against *S. aureus* ATCC 43300 **(A)** and ATCC 29213 **(B)**. *S. aureus* strains were treated with 0.5× MIC, 1× MIC, 2× MIC of OB and DMSO with PBS (solvent control), respectively. Samples were taken at 0, 1, 2, 3, 4, 5, 6, 12, and 24 h, and the number of viable bacteria was counted as CFU/ml. The data were presented as mean ± SD. Multi-step resistance selection of *S. aureus* ATCC 43300 **(C)** and ATCC 29213 **(D)** to OB and the antibiotic ciprofloxacin. The value of MIC_*n*_/MIC_1_ represents the average fold changes of MIC according to the MIC of the first passage.

To investigate whether the bactericidal activity of OB on *S. aureus* ATCC 43300 and ATCC 29213 was in a time-dependent and dose-dependent manner, time-kill assays were conducted (Figures 2A,B). Bactericidal activity was observed when *S. aureus* ATCC 43300 and ATCC 29213 were treated with 2× MIC of OB. No viable bacteria were observed after 5 h. Treatment with 1× MIC of OB also inhibited the growth of *S. aureus* ATCC 43300 and ATCC 29213, with a reduction in viable cell counts by 5-log10 and 4-log10 after 24 h, respectively.

### Antimicrobial Activities of OB Against ESKAPE Pathogens

The MICs of OB against *E. faecalis* ATCC 29212 and *S. epidermidis* RP62A were 16 and 8 μg/ml, respectively (data not shown), whereas the MICs for Gram-negative bacteria were higher (ranging from 16 to 128 μg/ml), indicating that OB had greater antibacterial effect on Gram-positive bacteria. To improve the antibacterial potency of OB on Gram-negative bacteria, an outer membrane–disrupting antibacterial peptide PMBN was used to permeabilize the cells. The checkerboard assay was designed to evaluate the effect of OB on Gram-negative bacteria in combination with PMBN (Table 2). All Gram-negative members of the ESKAPE pathogens were insensitive to PMBN (MICs > 128 μg/ml). However, when OB was combined with PMBN (32 μg/ml), the MIC of OB against *K. pneumoniae* ATCC 700603 was decreased from 128 to 16 μg/ml and the MIC of OB against *P. aeruginosa* ATCC 15692 was decreased from 64 to 8 μg/ml. When the FICIs were calculated, OB showed significant synergistic effects with PMBN against *K. pneumoniae*, *P. aeruginosa*, and *E. coli* (FICI < 0.375); additive effect was also observed against *A. baumannii* (0.5 < FICI < 0.75).

**TABLE 2:** Antimicrobial activities of OB and PMBN against Gram-negative bacteria.

**Strains**	**MIC_*OB*_ (μg/ml)**		**MIC_*PMBN*_ (μg/ml)**		**Outcome (FICI)**
	**Alone**	**Combination**	**Alone**	**Combination**	
*K. pneumoniae* ATCC 700603	128	16	>128	32	Synergy (<0.375)
*A. baumannii* ATCC 1195	16	8	>128	32	Addition (<0.75)
*P. aeruginosa* ATCC 15692	64	8	>128	32	Synergy (<0.375)
*E. coli* ATCC 25922	64	4	>128	16	Synergy (<0.1875)

### Anti-biofilm Activities of OB Against *S. aureus*

**FIGURE 3:**
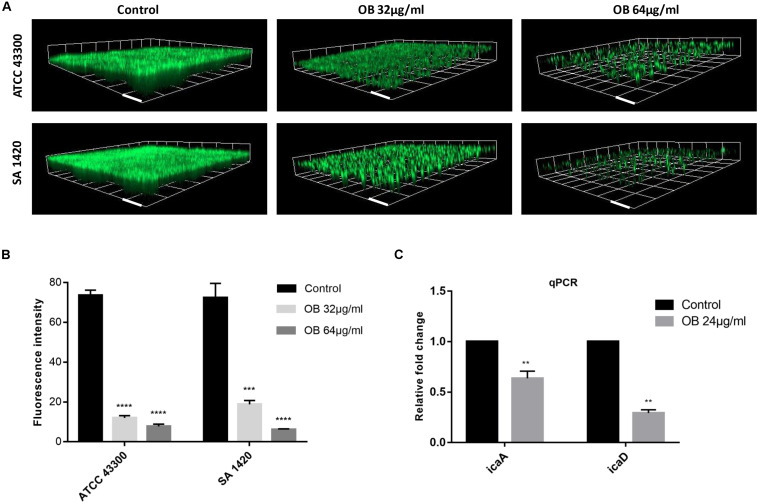
The anti-biofilm activities of OB against *S. aureus*. **(A)** Three-dimensional CLSM images of biofilm formed on glass slides using the fluorescence dye SYTO9. The mature biofilm formed by *S. aureus* ATCC 43300 and SA 1420 (clinical MRSA strain) was treated with DMSO (solvent control), 32 or 64 μg/ml of OB for 24 h. The images were processed with Zen 3.0. Scale bars, 20 μm. **(B)** Corresponding quantitative histogram of fluorescent intensity detected by CLSM. Compared with control group, ***P < 0.001;****P < 0.0001 (unpaired two-tailed Student’s *t*-test). **(C)** Relative expression of *icaA* and *icaD*. *S. aureus* ATCC 43300 was cultured with or without OB (24 μg/ml) for 24 h, and the transcription of *icaA* and *icaD* were measured by qPCR. Compared with control group, **P < 0.01(unpaired two-tailed Student’s *t*-test).

The biofilm-forming capacity of all *S. aureus* strains are shown in Supplementary Table S2 with strong biofilm producers selected for the following experiments. The eradication effect of OB on mature biofilm was illustrated (Table 1). Oxacillin had no obvious anti-biofilm effect on most of the tested isolates (MBEC_50_ > 128 μg/ml). Although vancomycin had potent antibacterial activity against MRSA, it did not eradicate biofilm obviously, and there were only three tested isolates with MBEC_50_ of 16 μg/ml. However, OB was able to eradicate biofilm partially with MBEC_50_ of 16–64 μg/ml, and the eradication rate for certain MRSA strains reached 80%. The CLSM also showed that as the concentration of OB increased, the green fluorescence signal (viable bacteria stained green) was significantly weakened, and the thickness and density of the biofilm was decreased (Figure 3A), which was consistent with the results of quantitative analysis (Figure 3B). There is a research reported that hydroxypropyltrimethyl ammonium chloride chitosan inhibited the expression of *icaA*, which mediates the production of extracellular polysaccharides, both in new biofilms and in pre-existing biofilms on titanium (Peng et al., 2011). As a quaternary ammonium derivative, OB may also affect the expression of some biofilm-related genes. The qPCR analysis results showed that compared with the control group, the relative expression of *icaA* and *icaD* genes decreased after treatment with 24 μg/ml of OB (*P* < 0.01) (Figure 3C).

### Resistance Selection of OB for *S. aureus*

The results of multi-step resistance selection are shown in Figures 2C,D. When *S. aureus* ATCC 43300 and ATCC 29213 were cultured in the presence of sub-inhibitory concentration of ciprofloxacin, the MICs increased 20- and 12-fold after 20 passages, respectively. However, the MICs of OB almost did not change from the first passage to the last passage. For single-step resistance selection (Table 3), the spontaneous mutation frequency to OB was lower than comparator agents like rifampicin and ciprofloxacin. The spontaneous mutation frequency of *S. aureus* ATCC 43300 and ATCC 29213 treatment with 4× MIC of OB were < 5.92 × 10^–10^ (± 8.37 × 10^–10^) and < 3.81 × 10^–10^ (± 5.39 × 10^–10^), whereas treatment with 4× MIC of rifampicin were 9.81 × 10^–9^ (± 4.80 × 10^–9^) and 6.62 × 10^–9^ (± 1.69 × 10^–9^), respectively. In summary, regardless of the long-term or short-term process of *in vitro* drug-resistance evolution, OB had a greater advantage over rifampicin and ciprofloxacin in reducing the production of resistant colonies.

**TABLE 3:** Single-step resistance selection of OB for *S. aureus*.

**Strains**	**Antimicrobial**	**Spontaneous resistance frequency (± SD)**	
		**2× MIC**	**4× MIC**
ATCC 43300	OB	<6.35 × 10^–10^ (± 8.98 × 10^–10^)	<5.92 × 10^–10^ (± 8.37 × 10^–10^)
	Rifampin	1.43 × 10^–8^ (± 6.26 × 10^–9^)	9.81 × 10^–9^ (± 4.80 × 10^–9^)
	Ciprofloxacin	1.05 × 10^–7^ (± 5.95 × 10^–8^)	<3.92 × 10^–10^ (± 5.55 × 10^–10^)
ATCC 29213	OB	<4.94 × 10^–10^ (± 6.98 × 10^–10^)	<3.81 × 10^–10^ (± 5.39 × 10^–10^)
	Rifampin	7.70 × 10^–9^ (± 3.06 × 10^–9^)	6.62 × 10^–9^ (± 1.69 × 10^–9^)
	Ciprofloxacin	9.25 × 10^–9^ (± 5.37 × 10^–9^)	< 3.17 × 10^–10^ (± 4.49 × 10^–10^)

### Effect of OB on Membrane Permeability

**FIGURE 4:**
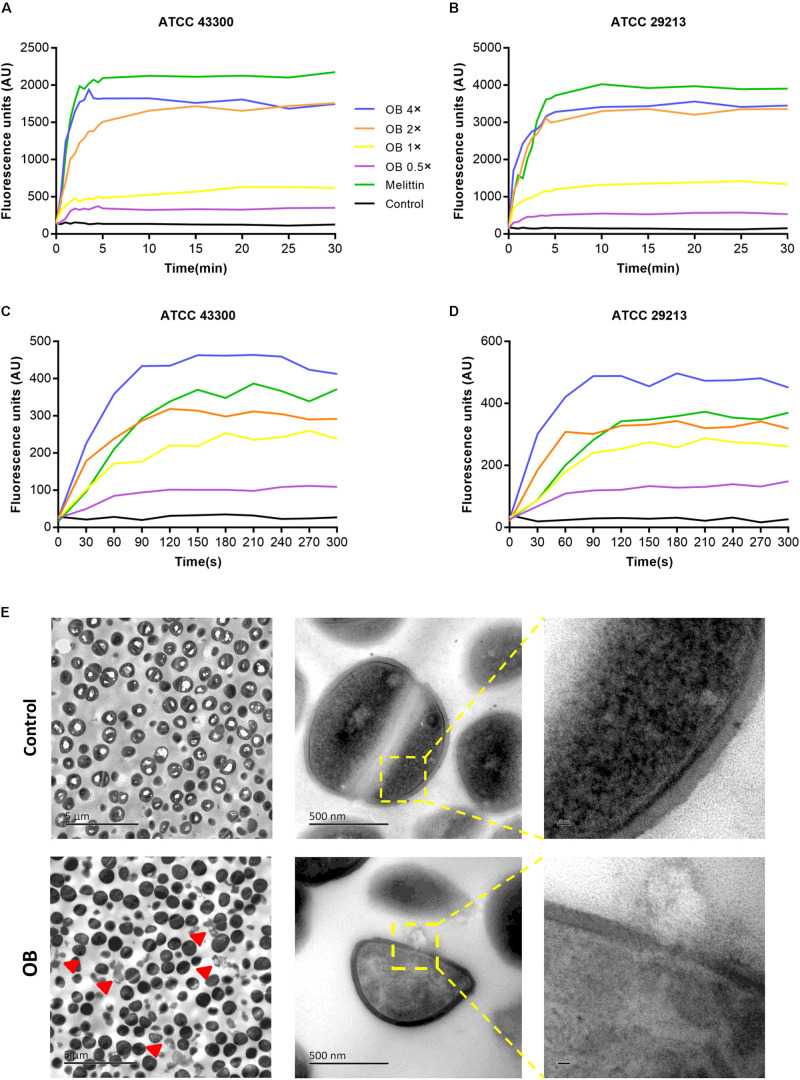
Effect of OB on membrane permeability. *S. aureus* ATCC43300 and ATCC29213 were incubated with 0.5× MIC, 1× MIC, 2× MIC, 4× MIC of OB, and the effect of OB on the fluorescence intensity of Disc3(5) **(A,B)** and SYTOX Green **(C,D)** were detected. 20 μg/ml melittin was used as a positive control and 0.1% DMSO with HEPES was a negative control. **(E)** The TEM images of *S. aureus* ATCC43300 exposed to DMSO with PBS (solvent control) or 64 μg/ml of OB. Red arrowheads show areas of cytoplasmic leakage. Scale bars, 5 μm (left), 500 nm (middle), and 20 nm (right).

To further investigate the antibacterial mechanism of OB against *S. aureus*, the nucleic acid dye SYTOX Green was used to detect the integrity of the cell membrane. Treatment with varying concentrations of OB on *S. aureus* ATCC 43300 and ATCC 29213 caused a dose-dependent increase in fluorescence intensity in 5 min, and the fluorescence signal did not decrease in the next 25 min (Figures 4A,B). A similar phenomenon was observed using the membrane potential sensitive dye Disc3(5). Treatment with 4× MIC of OB to *S. aureus* strains resulted in an increase in fluorescence intensity (400–450 AU) within 300 s (Figures 4C,D). Melittin as a positive control has been confirmed to have strong membrane lytic property, so the enhancement of fluorescence signal could also be detected. Together, these results demonstrated that the antimicrobial mechanism of OB was likely similar to melittin which was able to combine within the cell membrane and cause membrane damage.

To visually observe bacterial membrane disruption by OB, transmission electron microscopy (TEM) was employed. *S. aureus* ATCC 43300 (2 × 10^9^ CFU/ml) was incubated with 64 μg/ml of OB (which was a sub-inhibitory concentration for this high inoculum, no statistical difference in the number of viable bacteria before and after treatment, from 2.57 ± 0.74 × 10^9^ to 1.43 ± 0.17 × 10^9^, *P* < 0.05, unpaired two-tailed Student’s *t*-test) for 1 h at 37°C. The TEM showed that the boundary of cell membrane became blurred after treatment and intracellular contents were observed to leak from the cell. In contrast, the control group showed that the cell membrane was unaffected. Therefore, these results further demonstrated that OB targets the membrane (Figure 4E).

Because OB also showed antimicrobial activity against Gram-negative bacteria with MICs of 16–128 μg/ml, we hypothesized that OB probably achieved this effect by acting on the inner membrane of Gram-negative bacteria. To detect the inner membrane integrity, the ONPG experiment was performed with *E. coli* as an example. *E. coli* ATCC 29212 incubated with OB caused a dose-dependent and time-dependent increase in optical density value (A_405 *nm*_). In treatment with 2× MIC of OB for 1.5 h, the optical density value increased from 1.3 to 1.6. In contrast, no increase of optical density value was detected according to the control group (Supplementary Figure S1).

### Hemolytic Activity and Cytotoxicity

The results of hemolysis assay and cytotoxicity test are shown in Supplementary Figure S2. The HC_50_ of OB for human RBCs was 51.67 μg/ml, whereas the IC_50_ value of OB for human breast cancer cell lines KPL-4 and human colonic epithelial cell NCM-460 were 36.66 and 42.61 μg/ml, respectively. The MICs and MBCs of OB was lower than the IC_50_ and HC_50_ values of OB, suggesting that OB had great antimicrobial activity and low toxicity.

### Therapeutic Efficacy of OB in MRSA-Infected Mouse Peritonitis Model

Before *in vivo* efficacy testing, toxicity of OB was evaluated in mice (Supplementary Figure S3). Intraperitoneal injection of OB with a dose of 40 mg/kg twice was well-tolerated in all mice, with no death observed up to 7 days post-injection, and there was almost no change in body weight compared with the vehicle group, indicating that this dose is relatively safe for treatment of peritonitis. When the OB was used consecutively for 7 days, no death was observed but the weight of mice decreased slightly compared with the vehicle group. For blood cell analysis, there was no statistical difference among the parameters (Supplementary Table S3). For histological analysis, the results showed that OB did not cause significant injury on the tissues, but there were mild morphological changes with hepatocytes in the liver and a little hemorrhagic focus in the kidney as compared with the vehicle group.

**FIGURE 5:**
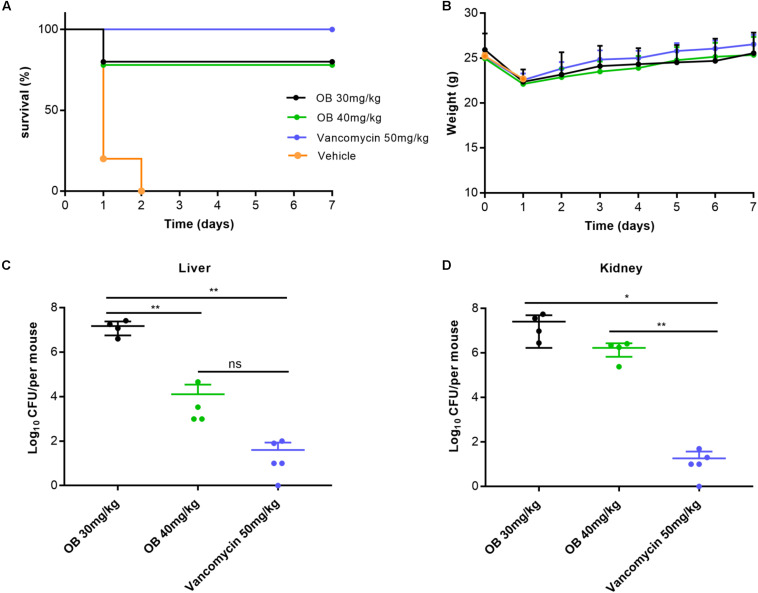
Therapeutic efficacy of OB *in vivo*. Groups of mice (*n* = 5) were given 30 mg/kg of OB, 40 mg/kg of OB, 50 mg/kg of vancomycin (positive control), or saline with 2% DMSO (vehicle) at 1 and 5 h post-infection. The survival condition **(A)** and weight changes **(B)** of the infected mice were observed for 7 days. The number of viable bacteria in liver **(C)** and kidney **(D)** of the survived mice after 7 days were counted. The data were presented as mean ± SD. *P < 0.05; **P < 0.01 (one-way ANOVA, Dunnett’s multiple-comparison test).

The antibacterial activity *in vivo* was evaluated by MRSA-infected mouse peritonitis model. The mice (*n* = 5) in vehicle group were intraperitoneally injected with (2–3) × 10^8^ CFU/ml of MRSA (500 μl), which resulted in a 100% mortality rate within 48 h. Therefore, the inoculation amount of MRSA used in this model was a lethal dose that could lead to acute peritonitis. Compared with the vehicle group, treatment with 30 or 40 mg/kg of OB at 1 and 5 h post-infection cured 80% of mice, whereas treatment with 50 mg/kg of vancomycin (positive control) cured 100% of mice (Figure 5A). The weight of all mice decreased slightly on the first day after infection and then increased to the initial value after 7 days (Figure 5B). The mice in the vehicle group died within 48 h, and the number of viable bacteria in liver and kidney was estimated to determine the actual amount of infected bacteria. The bacterial loads of MRSA in liver and kidney were approximately 2.5 × 10^8^ and 5.2 × 10^7^CFU/mouse (data not shown), which is in accordance with the initial injection amount. Moreover, the number of viable bacteria in liver and kidney of the survived mice was also counted. Only a few bacteria could be detected in liver or kidney of the mice treated with 50 mg/kg of vancomycin. There was still a certain amount of bacteria in the liver or kidney of the mice treated with OB, but there was no statistical difference in the number of bacteria in liver of the mice treated with 40 mg/kg of OB and 50 mg/kg of vancomycin. Compared with the group treated with 30 mg/kg of OB, the number of viable bacteria in liver exhibited a 3-log10 reduction in the group treated with 40 mg/kg of OB (*P* < 0.01) (Figures 5C,D).

## Discussion

In this report, we found that compared with Gram-negative bacteria, OB had stronger antibacterial activity against Gram-positive bacteria, which might be a result of the differences in the composition and structure of the cell wall. The cell wall structure of Gram-negative bacteria is more complicated, and its unique component outer membrane has selective permeability to molecules, thus reducing the sensitivity of bacteria to certain drugs. PMBN had no obvious antibacterial activity as well as bactericidal activity, but it increased the permeability of the outer membrane and promoted the entry of OB into the Gram-negative bacteria (Tsubery et al., 2000). Therefore, the application of PMBN improved the antibacterial efficiency of OB and expanded its antibacterial spectrum. As we know, biofilm is the structured communities of bacteria and is more resistant to antibiotic treatment (Coenye and Nelis, 2010). Our results showed that OB had a stronger effect on biofilm eradication than vancomycin, which reflects its superiority as an antibacterial agent in the treatment of biofilm-related infections. In addition, it could also reduce the expression of *icaA* and *icaD* genes which mediated the synthesis of polysaccharide intercellular adhesin, the main exopolysaccharide component of staphylococcal biofilm matrix (Arciola et al., 2015).

Quaternary amine compounds (QACs) are widely used in clinical disinfection to prevent the spread of bacteria and act through membrane disruption. The positively charged head group in compounds can be attracted by the negative charge on the bacterial surface to form an electrostatic bond, which increases the pressure on the cell wall and promotes the penetration of long-chain alkyl into the cell membrane, and eventually leads to the leakage of intracellular contents and cell death (Oblak et al., 2019). Because OB has similar chemical features, we speculated that it likely also disrupts the cell membrane. Therefore, we performed the assay using fluorescent dyes and found that OB could depolarize the cell membrane and increase its permeability. Subsequently, we microscopically observed that the integrity of cell membrane was destroyed after treatment with OB. ONPG experiment also showed that OB could penetrate the inner membrane of Gram-negative bacteria. Moreover, membrane-targeting antimicrobials reconfigure the membrane and interfere with the basic function of bacterial membrane, which makes it extremely hard for bacteria to survive and develop drug resistance (Koh et al., 2016; Lin et al., 2017). Our result also showed that OB had rapid bactericidal ability and a low tendency for resistance development, which further confirmed our hypothesis that OB was a membrane-targeting agent.

In addition to its membrane-disrupting activity, several researches propose that QACs may also affect other cellular components and this contributes to their antimicrobial action. Bore et al. (2007) reported that benzalkonium chloride treatment might result in superoxide stress in *E. coli* K-12. Knauf et al. (2018) suggested that benzalkonium chloride might act directly on the ribosome or influence ribosome association with other important proteostasis components to induce accumulation of protein aggregates in *A. baumannii*. Because OB has a similar scaffold, we hypothesize it is possible that OB has very specific targets within the bacterial cell to induce cell death, but its binding site and molecular mechanism need to be further investigated.

OB has no obvious side effects and slight toxicity. OB can neither affect the gastric secretion nor produce atropine-like side effects during treatment in patients with IBS (Heading et al., 2006). Acute toxicity experiments revealed that the non-lethal dose for oral administration was 1500 mg/kg for rats and 1000 mg/kg for dogs. In the chronic toxicity experiment, a dose of 80 mg/kg of OB was orally administered to the experimental animals for 180 days, and no change was found in blood chemistry or histologic profiles (Triantafillidis and Malgarinos, 2014). Our data also showed that OB had low hemolysis rate and limited cytotoxicity. The HC_50_ and IC_50_ values are significantly higher than the MIC and MBC values, indicating that the recommended antibacterial dose is likely safe. In addition, in *in vivo* toxicity testing, OB did not cause mice death and significant injury on tissues after treatment with 40 mg/kg of drug by intraperitoneal injection for 7 days. In MRSA-infected mouse peritonitis model, although the weight of survived mice reduced after infection, it could return to the initial level after treatment with OB, which also illustrated that the toxicity of OB was slight.

In the present study, we used mouse peritonitis model to evaluate the therapeutic effect of OB *in vivo*. This model was chosen because it is easy to master, the end points are clear (death or survival), and the model has been validated with marketed antibiotics (Ford et al., 1996; Bouley et al., 2016). However, OB has poor systemic absorption and primarily remains in the gastrointestinal tract after oral administration. Pharmacokinetic studies revealed that the drug is rapidly eliminated from the circulation within 4 h (Evangelista, 1999). To improve the utilization of the drug and avoid gastrointestinal side reactions, intraperitoneal route of administration was used in this model. There are abundant capillaries on the peritoneum of mice, which can increase the absorption area of the drug and accelerate its circulation (Al Shoyaib et al., 2020). Through animal experiment, we found that OB was effective *in vivo* and improved the survival rate of mice. However, it is still worth exploring how to optimize pharmacologic properties and reduce toxicity of OB for treatment of *S. aureus* infections more efficiently. This may include drug combination, new chemical analogs, and nanoscale carriers (Pham et al., 2019).

In summary, OB not only has strong antibacterial ability against *S. aureus* but also has a potent effect on eradicating mature biofilm. Furthermore, it lacks resistance development and has low cytotoxicity. *In vivo*, OB is effective for peritonitis infection model caused by MRSA. All these findings emphasize the potential of OB for further clinical development.

## Data Availability Statement

All datasets generated for this study are included in the article/Supplementary Material.

## Ethics Statement

Clinical samples collection and animal experiments were conducted under the approval of the Ethics Committee of the Third Xiangya Hospital, Central South University (Nos. 2019sydw0233 and 2019-S021). Strains and blood were isolated from clinical samples routinely collected from patients, and the identification of patients was not needed. Therefore, the need for written informed consent was waived and oral informed consent was obtained.

## Author Contributions

YW, PS, and LZ designed the experiments. LZ performed most of the experiments, analyzed the results, and wrote the article. LC, ZL, and FT provided essential reagents and methods. SL and XZ performed the supporting experiments. YW conceived and supervised the study. All authors contributed to the article and approved the submitted version.

## Conflict of Interest

The authors declare that the research was conducted in the absence of any commercial or financial relationships that could be construed as a potential conflict of interest.

## Supplementary Material

FIGURE S1 Effect of OB on inner membrane permeability. *E. coli* ATCC 25922 was incubated with 0.5× MIC, 1× MIC, 2× MIC of OB or DMSO (solvent control), and the absorbance at 405 nm were monitored with ONPG for 1.5 h. The data were presented as mean ± SD.

FIGURE S2 Hemolytic activity and cytotoxicity of OB. **(A)** 4% (*v*/*v*) human RBCs were treated with serially diluted OB at 37°C for 1 h, and the absorbance of the supernatants was measured at 540 nm to calculate the hemolysis (%). 0.1% DMSO with PBS was a negative control, and 0.1% Triton X-100 was a positive control. Human breast cancer cell line KPL-4 **(B)** and human colon epithelial cell NCM-460 **(C)** were treated with OB for 24 h, 10 μl of CCK-8 was added and the absorbance at 450 nm was recorded to calculate the cell viability (%). The data were presented as mean ± SD.

FIGURE S3 *In vivo* toxicity of OB. **(A)** Weight changes of mice treated with 40 mg/kg of OB twice (i.p) and observed for 7 days. Weight changes **(B)** and H&E staining **(C)** of the mice treated with 40 mg/kg of OB daily for consecutive 7 days (i.p.) and observed for 12 days. Scale bars, 50 μm.
