# Supplementary figures and images for: Repurposing Antispasmodic Agent Otilonium Bromide for Treatment of Staphylococcus aureus Infections
Source: Front Microbiol. 2020 Jul 31;11:1720. doi: 10.3389/fmicb.2020.01720 (PMC7410927; doi:10.3389/fmicb.2020.01720)

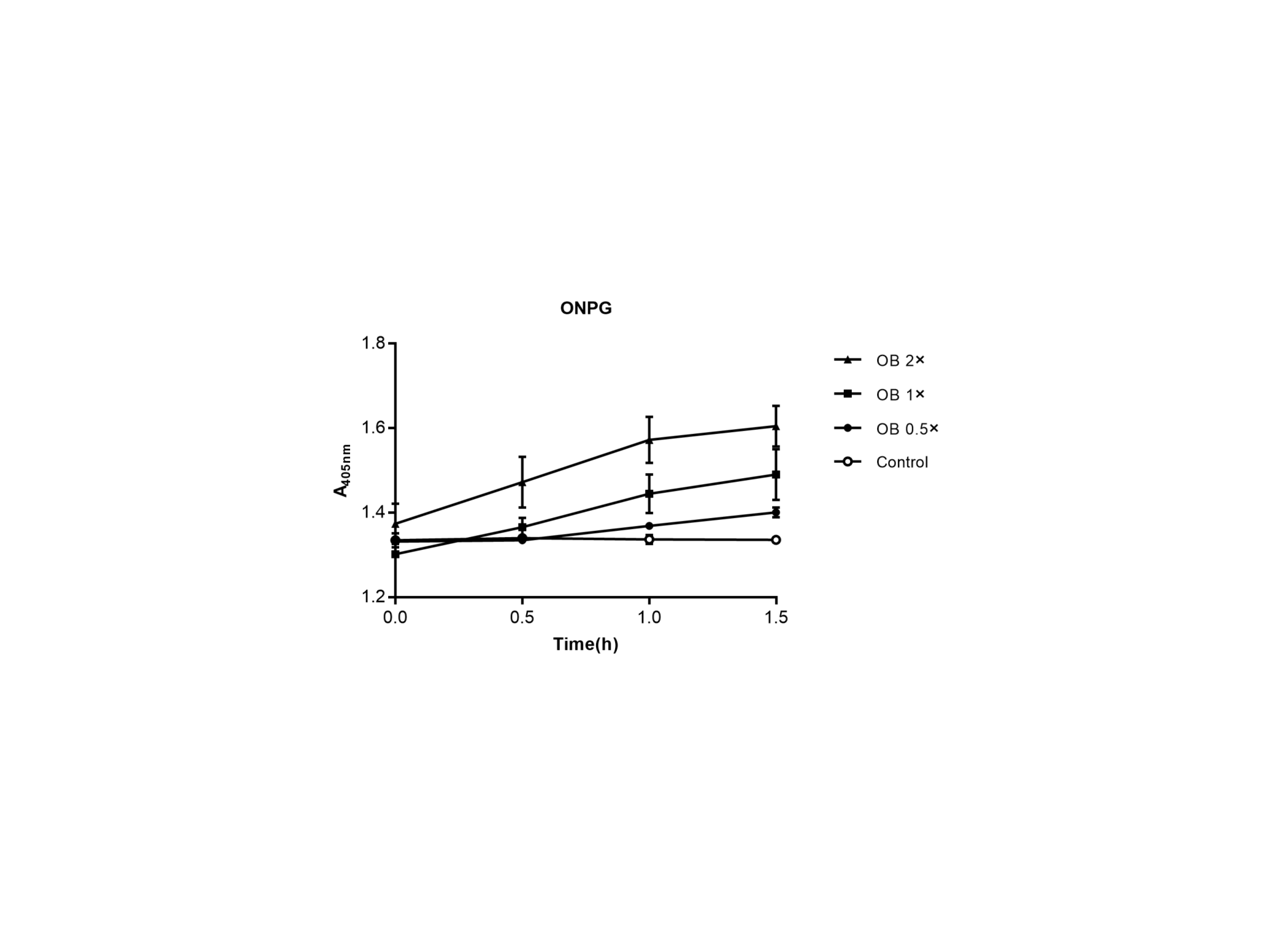

Supplement: FIGURE S1 — Effect of OB on inner membrane permeability. E. coli ATCC 25922 was incubated with 0.5× MIC, 1× MIC, 2× MIC of OB or DMSO (solvent control), and the absorbance at 405 nm were monitored with ONPG for 1.5 h. The data were presented as mean ± SD. [file Image_1.tif]

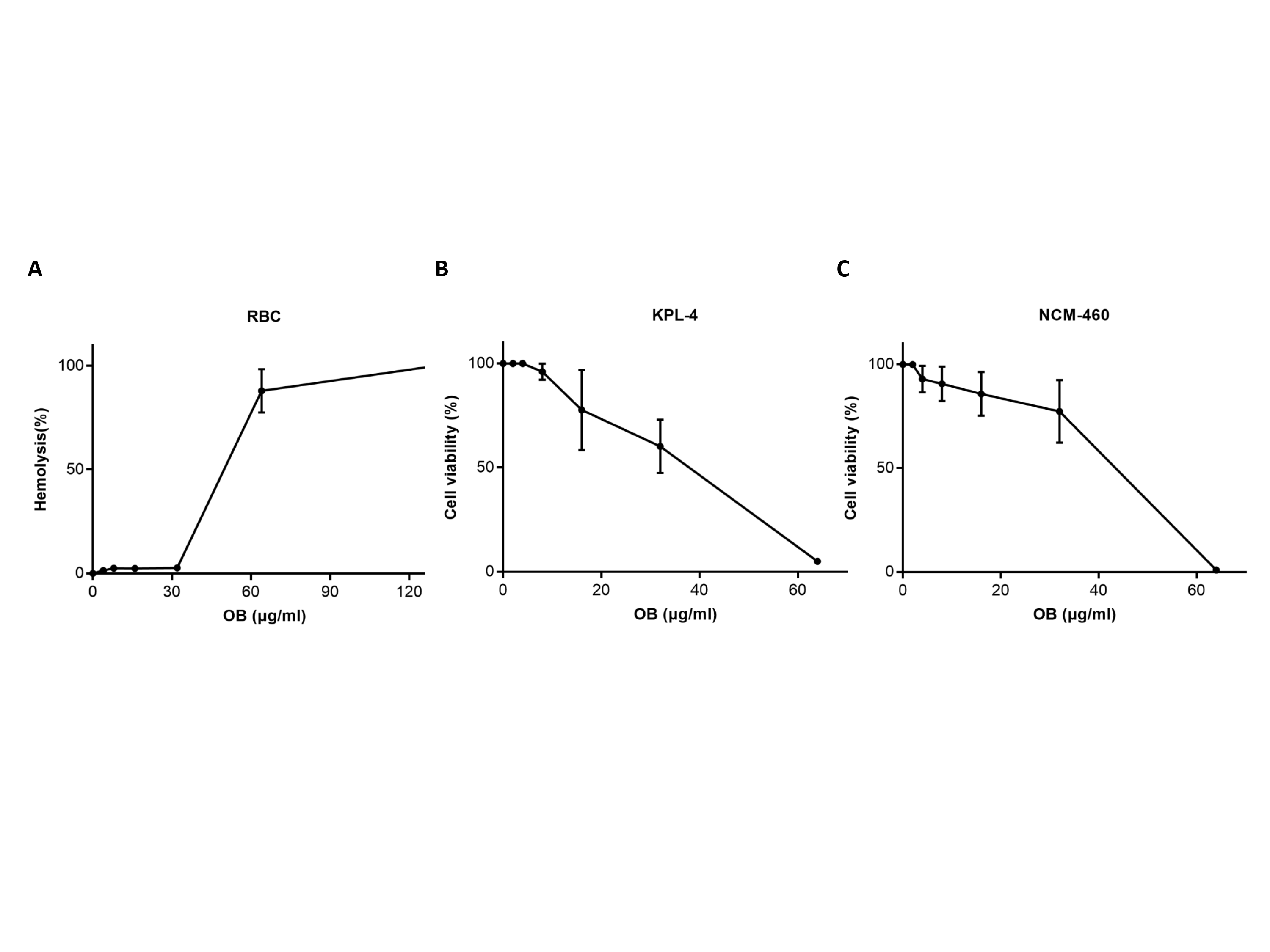

Supplement: FIGURE S2 — Hemolytic activity and cytotoxicity of OB. (A) 4% (v/v) human RBCs were treated with serially diluted OB at 37°C for 1 h, and the absorbance of the supernatants was measured at 540 nm to calculate the hemolysis (%). 0.1% DMSO with PBS was a negative control, and 0.1% Triton X-100 was a positive control. Human breast cancer cell line KPL-4 (B) and human colon epithelial cell NCM-460 (C) were treated with OB for 24 h, 10 μl of CCK-8 was added and the absorbance at 450 nm was recorded to calculate the cell viability (%). The data were presented as mean ± SD. [file Image_2.tif]

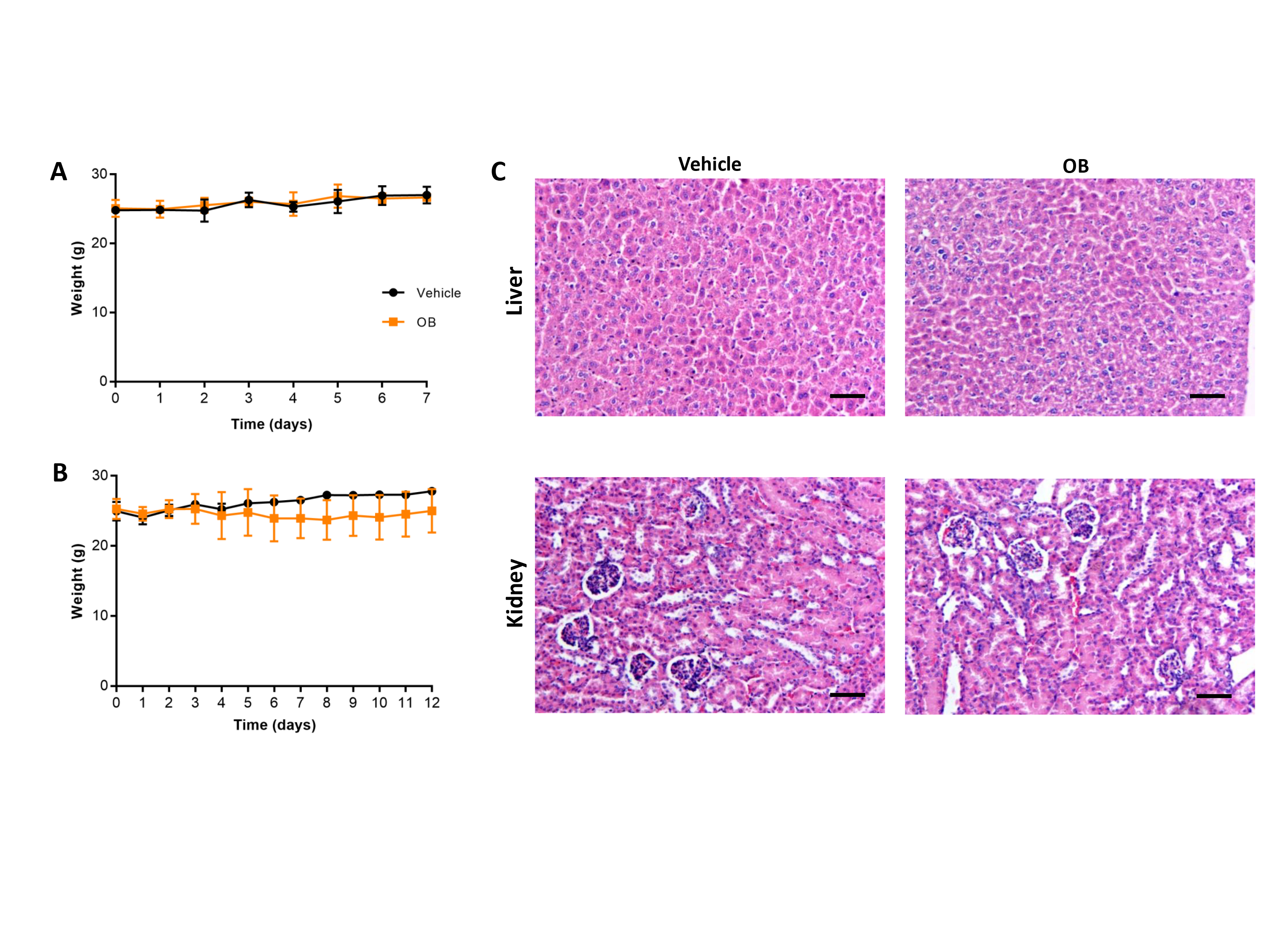

Supplement: FIGURE S3 — In vivo toxicity of OB. (A) Weight changes of mice treated with 40 mg/kg of OB twice (i.p) and observed for 7 days. Weight changes (B) and H&E staining (C) of the mice treated with 40 mg/kg of OB daily for consecutive 7 days (i.p.) and observed for 12 days. Scale bars, 50 μm. [file Image_3.tif]
