# Supplementary material for: Repurposing Antispasmodic Agent Otilonium Bromide for Treatment of Staphylococcus aureus Infections
Source: Front Microbiol. 2020 Jul 31;11:1720. doi: 10.3389/fmicb.2020.01720 (PMC7410927; doi:10.3389/fmicb.2020.01720)
Supplement: Supplementary file 4 [file Data_Sheet_1.docx]

**Supplementary data**

**Table S1 Classification of biofilm production**

| Average A_570nm_ value | Biofilm production |
| --- | --- |
| ≤Ac | Non |
| Ac < ~ ≤ 2🞩Ac | Weak |
| 2🞩Ac < ~ ≤ 4🞩Ac | Moderate |
| >4🞩Ac | Strong |

Absorbance cut-off value (Ac) = average A_570nm_ of negative control + 3🞩standard deviation (SD) of negative control.

**Table S2 Biofilm-forming capacity of *S. aureus***

| Strains | A_570nm_ | Biofilm production |
| --- | --- | --- |
| ATCC 43300 | 2.599 | Strong |
| ATCC 29213 | 0.927 | Moderate |
| Newman | 0.689 | Moderate |
| SA 1402 | 2.791 | Strong |
| SA 1405 | 1.59 | Strong |
| SA 1409 | 0.688 | Moderate |
| SA 1411 | 1.039 | Strong |
| SA 1415 | 3.484 | Strong |
| SA 1417 | 1.469 | Strong |
| SA 1419 | 0.791 | Moderate |
| SA 1420 | 1.74 | Strong |
| SA 1430 | 1.413 | Strong |
| LZ B1 | 3.34 | Strong |

Ac=0.234

**Table S3 Blood cells analysis of the mice treated with 40mg/kg of OB daily for consecutive 7 days**

| parameters | Vehicle | OB | *P* value |
| --- | --- | --- | --- |
| WBC (10^9^/L) | 6.53±2.65 | 9.00±2.13 | 0.277 |
| LYMPH# (10^9^/L) | 4.40±1.74 | 4.83±1.58 | 0.766 |
| MONO# (10^9^/L) | 0.37±0.06 | 0.77±0.25 | 0.060 |
| NEUT# (10^9^/L) | 1.77±0.95 | 3.40±1.13 | 0.127 |
| RBC (10^12^/L) | 8.82±0.32 | 8.41±1.24 | 0.614 |
| HGB (g/L) | 140.33±6.66 | 139.33±26.69 | 0.953 |
| HCT (%) | 46.53±2.01 | 45.17±6.87 | 0.757 |
| MCV (fL) | 52.83±0.65 | 53.73±2.24 | 0.540 |
| MCH (pg) | 15.83±0.21 | 16.43±0.99 | 0.361 |
| MCHC (g/L) | 301.00±1.00 | 306.67±13.20 | 0.500 |
| PLT (10^9^/L) | 1594.67±287.01 | 1798.00±212.88 | 0.380 |
| MPV (fL) | 5.17±0.12 | 6.70±1.21 | 0.095 |

The mice were treated with 40mg/kg of OB daily for 7 days (i.p.) and observed for 5 days after the final injection. Blood cell analysis was performed on the 12th day after the first injection. *P* values were calculated using multiple t tests.
